# Response guided therapy for reducing duration of direct acting antivirals in chronic hepatitis C infected patients: a Pilot study

**DOI:** 10.1038/s41598-020-74568-x

**Published:** 2020-10-20

**Authors:** Ohad Etzion, Harel Dahari, David Yardeni, Assaf Issachar, Anat Nevo-Shor, Michal Cohen-Naftaly, Yaffa Ashur, Susan L. Uprichard, Orly Sneh Arbib, Daniela Munteanu, Marius Braun, Scott J. Cotler, Naim Abufreha, Ayelet Keren-Naus, Yonat Shemer-Avni, Orna Mor, Jayanah Murad, Victor Novack, Amir Shlomai

**Affiliations:** 1 Department of Gastroenterology and Liver Diseases, Soroka University Medical Center, 151 Rager Yitzhak Blvd, 84171 Beer-Sheva, Israel; 2 The Program for Experimental and Theoretical Modeling, Division of Hepatology, Department of Medicine, Stritch School of Medicine, Loyola University Chicago, 2160 S. First Ave, Maywood, IL 60153 USA; 3 Department of Medicine D and The Liver Institute, Rabin Medical Center, Beilinson Hospital, Petah-Tikva, Israel; 4 Medical Management Unit, Soroka University Medical Center, Beer-Sheva, Israel; 5 Laboratory of Clinical Virology, Soroka University Medical Center, Beer-Sheva, Israel; 6 Central Virology Laboratory, Ministry of Health, Sheba Medical Center, Ramat Gan, Israel; 7 Clinical Research Center, Soroka University Medical Center, Beer-Sheva, Israel; 8 Faculty of Health Sciences, Ben-Gurion University of the Negev, Beer-Sheva, Israel; 9 Sackler Faculty of Medicine, Tel-Aviv University, Tel-Aviv, Israel

**Keywords:** Hepatitis C, Hepatitis C virus

## Abstract

The advent of direct-acting antivirals (DAAs) has transformed the landscape of hepatitis C virus (HCV) management. We aimed to prospectively (real-time) evaluate the feasibility of using a response-guided therapy approach, based on mathematical modeling of early viral kinetics, to reduce the duration of DAAs therapy. Patients were treated with DAAs according to the physicians’ preference. HCV was measured at baseline and at day 2 and weeks 1, 2 and 4 after treatment initiation. The primary endpoint was the proportion of patients with sustained-virological response (SVR) at 12 and/or 24 weeks post-treatment. Twenty-nine patients (mean age 54 ± 16, 44% females, 73% with HCV genotype 1), were enrolled and all completed therapy. Treatment duration was shortened in 11 of the 29 patients (38%). SVR was achieved in 28 of the 29 patients (97%). Relapse occurred post treatment in a single case of a non-cirrhotic male with genotype 3, who was treated with sofosbuvir/velpatasvir for 6 weeks. Virus sequencing did not identify baseline or treatment emergent resistance associated substitutions. Real-time mathematical modeling of early HCV kinetics can be utilized for shortening DAAs duration in approximately 40% of patients without compromising treatment efficacy.

Clinical trial registration: ClinicalTrials.gov Identifier: NCT03603327.

## Introduction

Chronic hepatitis C (CHC) infection affects about 71 million people worldwide and is a major cause of liver related morbidity and mortality^1^. Treatment of CHC is directed at achieving sustained virological response (SVR), which is defined as undetectable HCV RNA levels 12 or 24 weeks following completion of anti-viral therapy^2^ and is associated with improved long-term clinical outcomes^3^. The advent of all-oral direct-acting antivirals (DAAs) has transformed the landscape of HCV therapy by allowing achievement of SVR with few, if any side effects, in over 90% of patients treated with various regimens for 8 or 12 weeks^4–7^. Unfortunately, the promise brought by DAAs has not yet translated to the WHO goal of viral hepatitis elimination by 2030, defined as a 90% reduction in incidence and 65% reduction in mortality related to chronic hepatitis B and C^8^. Reaching this goal will require a substantial scaling-up of HCV screening and linkage-to-care programs, as well as reduction of treatment cost, which currently make treatment inaccessible to many^9^. Because shortening duration of DAA therapy would provide cost-saving, this could facilitate the goal of HCV elimination, especially in resource limited countries.

Historically, on-treatment viral kinetics was utilized to predict treatment outcomes and for devising stopping rules to avoid futile treatment with interferon-based therapy^10,11^. With the exceptionally high proportion of SVR achieved with DAA, viral kinetics no longer predict treatment failure, but may be utilized to individualize duration of treatment (termed time to cure, TTC) needed to achieve SVR^12^, using mathematical modeling, thereby substantially cutting treatment costs while maintaining efficacy.

HCV decline under DAA therapy follows biphasic kinetics. The rapid first phase occurs within 12–48 h of treatment initiation and is then followed by a slower second phase of several days to weeks in which viral decline continues at a constant rate^13^. While HCV becomes unquantifiable or undetectable at levels below 10–15 IU/mL, HCV cure is assumed to be reached when there is less than one virus particle in the entire extracellular body fluid (cure boundary)^14,15^. Mathematical modeling of HCV kinetics can reproduce the biphasic viral decline under DAA therapy, and therefore can predict time-to-cure (TTC), the timepoint at which cure boundary is reached.

Indeed, several retrospective studies published by our group have shown that mathematical modeling of viral kinetics predicts TTC of less than 12 weeks in the majority of individuals treated with sofosbuvir-based, as well as other DAA regimens^12,16,17^. To date, however, prospective assessment using mathematical models to individualize the duration of DAA treatment in CHC has not yet been performed.

The goal of the current study was to evaluate the feasibility of using a mathematical modeling-based response-guided therapy (RGT) approach to individualize the duration of HCV DAA therapy.

## Methods

### Patient population and clinical data

This was an open label, single arm, prospective, pilot study recruiting up to 30 consecutive CHC patients eligible for DAA treatment. Enrollment was conducted at 2 tertiary care hospitals in Israel, Soroka Medical Center in Beer-Sheva and Rabin Medical Center, Beilinson campus, in Petah-Tikva. Both centers are part of Clalit Health Services, the largest health fund in Israel. Male and female patients with compensated liver disease were enrolled in the study if they were ≥ 18 years of age, had HCV genotype 1–4, with RNA viral load > 10^5^ IU/mL at screening and on at least one other occasion 6 months or more prior to enrollment and had an ALT < 10 times the upper limit of normal. Patients were excluded from the study if they were pregnant, had evidence of another cause of chronic liver disease, had decompensated liver disease, eGFR < 60 mL/min as calculated by the Cockroft-Gault equation, imaging findings suspicious for hepatocellular carcinoma or evidence of extra-hepatic malignancy (excluding basal cell or squamous cell carcinoma) in the 5 years preceding enrollment. Full eligibility criteria for this study are provided in the Supplementary File.

Liver fibrosis staging was performed by either transient elastography or FibroTest. Cirrhosis was defined by liver stiffness measurement of ≥ 12 kPa or by a Fibrotest score of ≥ 0.75.

The following clinical and virological variables were recorded: demographics (age, gender), body mass index, treatment regimen and duration, HCV genotype, baseline viral load, complete blood count, alanine aminotransferase, albumin, creatinine, bilirubin and International Normalized Ratio, HCV RNA levels at specific time points prior to, during, and post-treatment and stiffness measurement or FibroTest score measurements. All authors had access to the study data and reviewed and approved the final manuscript.

### Ethical statement

The study was approved by the institutional review boards of Soroka and Rabin Medical Centers and was conducted in compliance with the Declaration of Helsinki, Good Clinical Practice guidelines, and local regulatory requirements. All patients provided written informed consent.

### Treatment regimens

Patients enrolled in the study received one of four DAA regimes currently provided by the health system in Israel:

1. Daily fixed dose combination of Elbasvir 50 mg + Grazoprevir 100 mg (ELB/GRZ)
2. Daily fixed dose combination of Ledipasvir 90 mg + Sofosbuvir 400 mg (SOF/LED)
3. Daily fixed dose combination of Sofosbuvir 400 mg + Velpatasvir 100 mg (SOF/VEL).
4. Daily fixed dose combination of Glecaprevir 100 mg + Pibrentasvir 40 mg (GLE/PIB).

Treatment regimens were selected based on the treating physicians’ preference, in alignment with the EASL 2018 guidelines for the treatment of CHC.

### Mathematical modeling

HCV viral kinetics under DAA therapy was assumed to follow the standard biphasic model^18^:

1 $$\frac{dI}{dt}=\beta {T}_{0}V-\delta I$$

$$\frac{dV}{dt}=\left(1-\varepsilon \right)pI-cV$$

where *T*_*0*_ represents the number of target cells (i.e., hepatocytes), *I*, the number of infected cells and *V*, is the viral load in blood. Virus, *V*, infects target cells with rate constant *β*, generating productively-infected cells, *I*, which produce new virions at rate *p* per infected cell. Infected cells are lost at a rate *δ* per infected cell and virions are assumed to be cleared from blood at rate c per virion. DAA effect *ε* is defined as the therapy efficacy 0 ≤ *ε* ≤ 1 in blocking viral production/secretion.

### Parameter estimations

Similar to our previous modeling studies, we assumed the target cell (i.e., hepatocytes) level remained constant during therapy at pre-treatment (or baseline) level *T*_*0*_ = *1* × *10*^*7*^. The initial infected cell level is represented by the steady state pre-treatment level of I_0_ = βV_0_T_0_/*δ*, where V_0_ = pre-treatment viral load of each patient. Viral production rate constant was set to p = cV_0_/I_0_. Because of lack of frequent sampling during the first 2 days after initiation of therapy the pharmacological delay of DAA was fixed to 0 h. Parameter β was set to 2 × 10^–7^ ml/virion/day. The remaining parameters (c, ε and *δ*) were estimated by fitting the model with the observed data using Berkeley Madonna (V.8.3).

### Time to cure

As previously done^12,14,16,17^, the TTC was defined as the time to reach less than one HCV particle in the entire extracellular body fluid, which was estimated based on body weight. For example, a value of 1 virus copy in 15L of extracellular body fluid volume, i.e. *V* = 7 × 10^–5^ IU/ml, was used as the threshold for cure. The model (Eq. 1) was fit to the measured HCV RNA kinetic data of each patient during the first 4 weeks of DAA therapy in real time in order to predict TTC for each participant.

### Patient monitoring and intervention

At baseline, blood was drawn for complete blood count, liver enzymes, bilirubin, albumin and HCV RNA. Following initiation of DAA therapy, repeated HCV RNA measurements were obtained on day 2, weeks 1, 2 and 4, at end-of-treatment (EOT) and then at weeks 4, 12 and/or 24 post-treatment. At week 4 of treatment, HCV RNA values from baseline through week 4 were modeled for each patient to project TTC. Based on individual model prediction of TTC, study participants were assigned the following lengths of DAA treatment:

1. Patients with estimated TTC  < 6 weeks of therapy – received a total of 6 weeks of DAA treatment.
2. Patients with estimated 6 ≤ TTC < 8 weeks of therapy- received a total of 8 weeks of DAA treatment.
3. Patients with estimated 8 ≤ TTC < 10 weeks of therapy- received a total of 10 weeks of DAA treatment.
4. Patients with estimated 10 ≤ TTC ≤ 12 weeks of therapy- received a total of 12 weeks of DAA treatment.
5. Patients with HCV RNA below lower limit of quantification (LLoQ) at day 2 – received a total of 6 weeks of DAA treatment.

Patients with estimated TTC of more than 12 weeks of therapy, with either ELB/GRZ, SOF/LED, or SOF/VEL, or of more than 8 weeks with PIB/GLE, received 12 or 8 weeks of DAA treatment, respectively, according to standard of care (SOC).

### Molecular assays

HCV RNA was measured by reverse transcription followed by real-time PCR using the GeneXpert assay (Cepheid, Sunnyvale CA, U.S) with a LLoQ of 10 IU/mL and lower limit of detection of 4 IU/mL. SVR was defined as undetectable serum HCV RNA 12 or 24 weeks after stopping antiviral treatment.

HCV genotyping was performed by the Abbott RealTime HCV Genotype II assay. This assay uses four sets of PCR primers. One set of primers targets a region within the 5′ untranslated region of the HCV genome that is recognized by GT-specific fluorescent-labeled probes. To subtype GT1, a second primer set is designed to amplify the nonstructural 5b region of genotype 1a and a third primer set is designed to amplify the nonstructural 5b region of genotype 1b (Abbott, Chicago, Illinois, USA).

In the single patient who relapsed, serum drug-resistant viral variants carrying resistant-associated substitutions (RAS) in the NS3 and NS5A regions, were explored by population (Sanger) sequencing^19^ (ABI PRISM 3100 genetic analyzer DNA Sequencer, Applied Biosystems, Foster City, CA, USA) and BigDye Terminator v1.1 Cycle Sequencing kit (Applied Biosystems, Foster City, CA, USA).

### Endpoints

The primary efficacy endpoints were: 1. The proportion of SVR at 12 or 24 weeks after EOT in all patients who received at least 4 weeks of therapy with any of the four DAA regimens. 2. The percentage of patients in whom duration of treatment with DAA could be shortened to less than SOC. Secondary endpoints included the development of treatment-associated RAS in patients who did not reach SVR, the rate of serious adverse events and treatment discontinuations due to adverse events.

### Statistical analyses

The primary efficacy analysis was designed to numerically compare the proportion of SVR among patients receiving RGT with one of the four DAA regimens, with a prespecified performance goal of 85%. This is a benchmark based on the general trend toward increasing proportions of SVR in recent years and was therefore chosen as a fixed, clinically relevant threshold representing a true measure of treatment benefit and a one to which the primary efficacy endpoint of this study was compared. This was a pilot study and therefore a formal power calculation of sample size was not applicable. We chose to enroll up to 30 patients to allow exploration of RGT efficacy.

The modified intention-to-treat population consisted of all patients who received at least 80% of their total HCV treatment drug doses throughout the entire treatment period and for whom HCV viral load data were available for baseline, day 2, week 1 and/or 2, week 4 and 12/24-week post-treatment. Patients who received less than 80% of the doses were to be excluded. This modified intention-to-treat population was used for analysis of the primary efficacy endpoints. This population was used for analysis of all safety endpoints.

Baseline medical histories and preexisting conditions were summarized by treatment duration groups. Viral load data was presented by treatment duration group and time point in summary tabulations and by-patient listings. These data were also presented graphically. Mean and median levels and change from baseline of the viral load was calculated at each time point. P-value < 0.05 was considered significant. Statistical analyses were performed using SPSS version 25 (IBM SPSS, Chicago, IL).

## Results

### Patient characteristics

**Figure 1:**
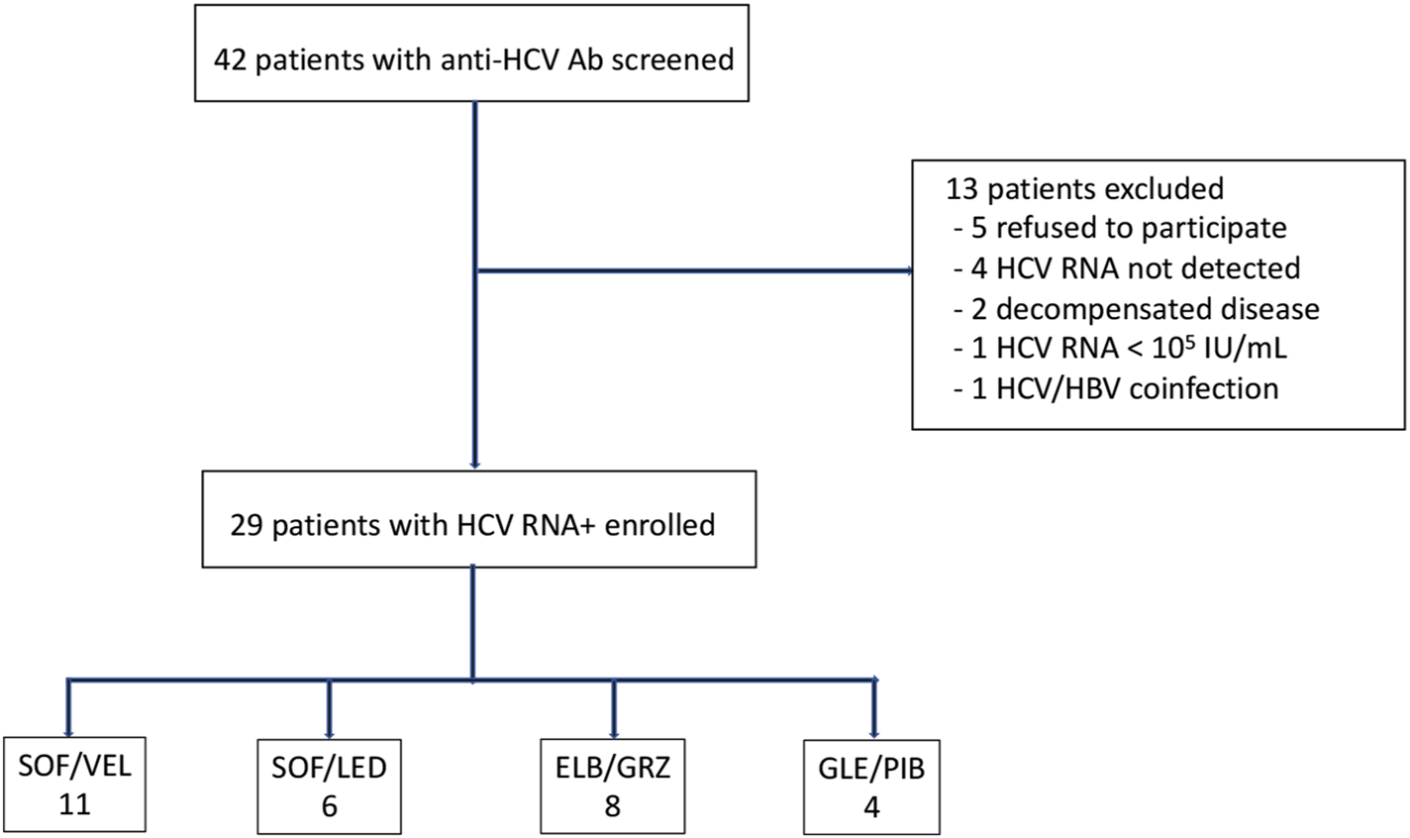
Study flow diagram. HBV, hepatitis B virus; HCV, hepatitis C virus; Ab, antibody; RNA, ribonucleic acid; IU, international units; mL, milliliters; SOF/VEL, sofosbuvir/velpatasvir; SOF/LED, sofosbuvir/ledipasvir; ELB/GRZ, elbasvir/grazoprevir; GLE/PIB, glecaprevir/Pibrentasvir.

Between February 2018 -April 2019, 42 patients were evaluated for eligibility. Of those, 13 were excluded due to the following reasons: 5 patients refused to participate, 4 patients had undetectable HCV RNA, one patient had a baseline HCV RNA < 10^5^ IU/mL, 2 patients had decompensated cirrhosis, and one patient had HBV coinfection. Overall, 29 patients (mean age 56, 44% females), with CHC-related compensated liver disease were enrolled to the study (Fig. 1). Baseline demographic, laboratory and virological features of study participants are summarized in Table 1. The most common genotype was 1b (66%), followed by genotype 3 (24%), genotype 1a (7%) and genotype 2 (3%). At baseline, 8 patients (27.6%) had evidence of advanced fibrosis or cirrhosis as per non-invasive tests. All patients but one, were naïve to anti-HCV therapy.

**Table 1:** Baseline demographic and clinical characteristics of study participants.

	Variable	Mean ± SD or N (%)
Demographics	Age (Mean ± SD)	53.5 ± 15.8
	Female, N (%)	13(44.8%)
	METAVIR 3/4, N (%)	8(27.6%)
Genotype N (%)	1B	19 (65.5%)
	1A	2 (6.9%)
	2	1 (3.5%)
	3	7 (24.1%)
Laboratory at baseline	ALT U/L (Mean ± SD)	56.1 ± 33.4
	Total Bilirubin mg/dL (Mean ± SD)	0.65 ± 0.5
	Albumin g/dL (Mean ± SD)	4.4 ± 0.3
	Hb g/dL (Mean ± SD)	14.2 + 1.4
	PLT Count 10^6^/mm^6^ (Mean ± SD)	53.5 ± 15.7
	HCV RNA IU/mL (Mean ± SD)	2.6 × 10^6^ ± 3.2 × 10^5^

SD, standard deviation; ALT, alanine aminotransferase; Hb, hemoglobin; PLT, platelets; HCV, hepatitis C virus; RNA, ribonucleic acid.

### Model-based administration of DAA treatment

DAA therapy with SOF/VEL, ELB/GRA, SOF/LED and GLE/PIB, was administered in 11, 8, 6 and 4 patients, respectively. Careful follow-up and frequent visits in the clinic confirmed that all patients who initiated treatment were fully adherent. Treatment was well tolerated across regimens with only five, grade I adverse events recorded (Supplementary Table 1).

**Figure 2:**
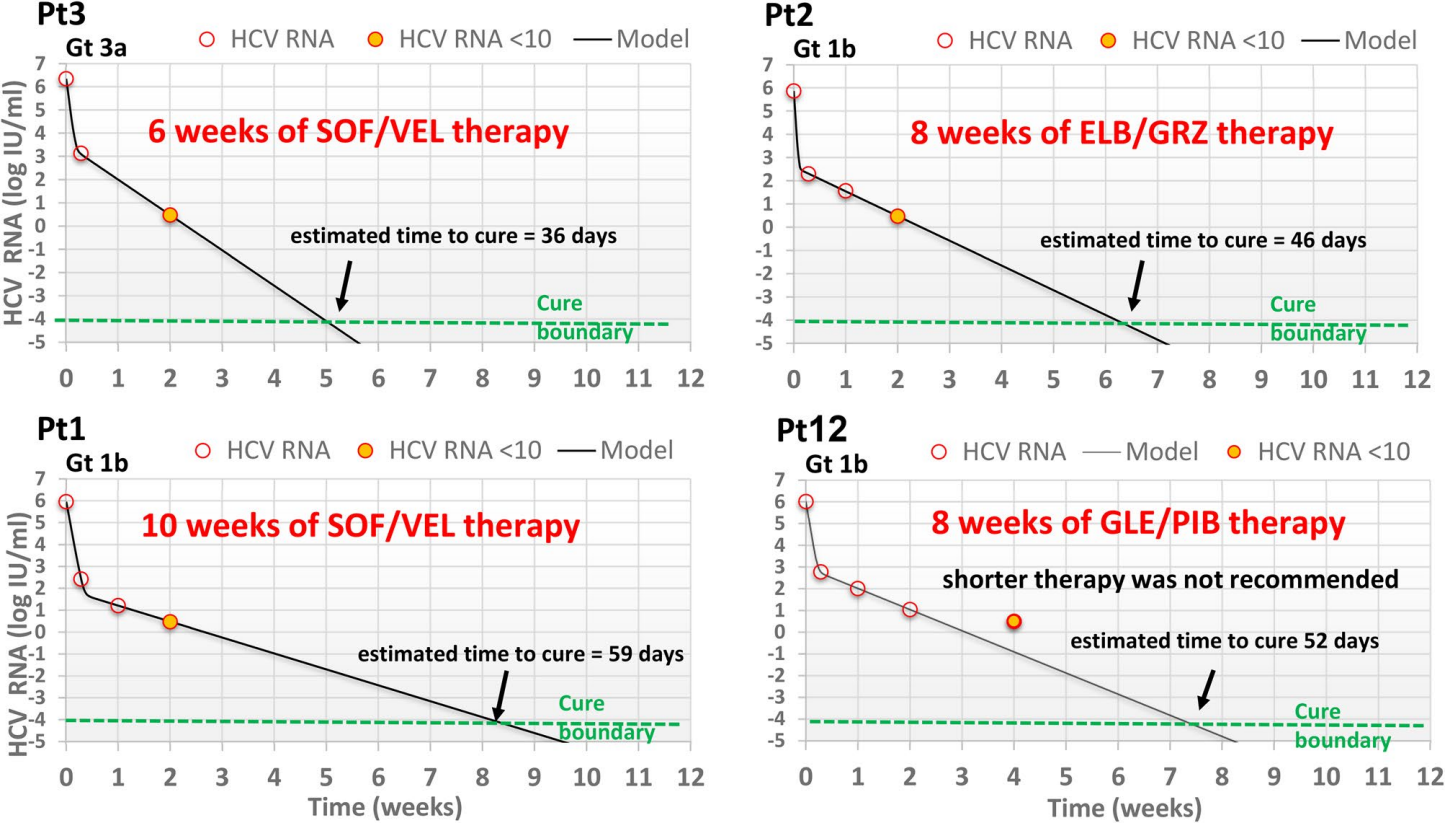
Modeling-based RGT approach in 4 representative patients. Gt, genotype; SOF/VEL, sofosbuvir/velpatasvir; SOF/LED, sofosbuvir/ledipasvir; ELB/GRZ, elbasvir/grazoprevir; GLE/PIB, glecaprevir/Pibrentasvir. Estimated viral kinetic parameters of patients in whom time to cure was predicted by modeling are shown in Table 2.

Of the 29 patients participating in the study, 25 (86.2%) were assigned to 12-week-based DAA regimens. In this group, mathematical modeling predicted TTC shorter than 12 weeks in 10 patients; 10 weeks in 1 patient, 8 weeks in 8 patients and 6 weeks in 1 patient (Fig. 2 and Table 2). Another patient for whom mathematical modeling was not feasible (reached viral load below LLoQ at day 2) was assigned to 6 weeks of therapy. The distribution of patients with shortened treatment duration according to DAA regimen is shown in Table 3. The other 14 patients in this group completed a full 12-week course of DAA therapy according to SOC. Modeling did not support reducing treatment duration below 8 weeks in any of the 4 patients treated with GLE/PIB. Baseline characteristics of patients predicted to reach cure with shortened DAA therapy were similar to those predicted to require full duration of treatment, except for baseline viral load which was significantly lower (p = 0.04) and ALT level which tended (p = 0.05) to be lower (Table 4). Only in the 11 patients in whom treatment was shortened, viral load at day 14 after initiation of therapy was < 15 IU/ml.

**Table 2:** Viral kinetic parameters and predicted time to cure of patients with shortened treatment.

Pt #	Sex	Age(yr)	Weight(kg)	HCV Gt	Fib	DAA regimen	V_0_*	c (d^-^^1^)	ε	δ (d^-^^1^)	Predicted TTC (d)	Actual DAA duration(w)
1	M	54	66	1b	F2	SOF/VEL	6.0	4.2	0.9999	0.25	59	10
2	M	61	104	1b	F3	ELB/GRZ	5.9	11.0	0.9995	0.35	46	8
3**	M	63	84	3a	F0-1	SOF/VEL	6.3	5.6	0.9988	0.5	36	6
4	F	29	55	1b	F0	SOF/LED	6.1	5.2	0.9993	0.25	55	8
5	M	59	71	3	F3	SOF/VEL	5.3	ND	ND	ND	ND	6
6	F	31	46	1B	F0-1	ELB/GRZ	5.2	5.7	0.9994	0.33	43	8
7	F	37	56	1A	F1	ELB/GRZ	6.3	11.2	0.9990	0.45	55	8
8	M	42	85	1B	F0-1	SOF/LED	6.3	4.0	0.9994	0.34	53	8
9	F	65	60	3	F2	SOF/VEL	5.0	7.3	0.9929	0.29	53	8
10	M	35	64	3A	F0-1	SOF/VEL	6.3	4.4	0.9993	0.41	44	8
11	F	67	80	1B	F1	ELB/GRZ	5.2	3.3	0.9999	0.28	53	8

*V_0_, initial viral load (log IU/ml) was set during fitting based on each patient’s measured baseline HCV RNA; **, relapser; Fib, Fibrosis stage; Gt, genotype; DAA, direct-acting antiviral; SOF/VEL, sofosbuvir/velpatasvir; SOF/LED, sofosbuvir/ledipasvir; ELB/GRZ, elbasvir/grazoprevir; TTC, Time to cure; ND, not determined because viral load was < 10 IU/ml at day 2 of therapy.

**Table 3:** Distribution of patients with shortened treatment according to DAA regimen.

DAA regimen	N	No. of patients in whom treatment duration shortened	% of total study patients in whom treatment duration shortened (%)	% of patients in whom treatment duration shortened per DAA regimen (%)	DAA-time saved (%)
SOF/VEL	11	5	46	46	17
SOF/LED	6	2	18	33	11
ELB/GRZ	8	4	36	50	17
GLE/PIB	4	0	0	0	
Total	29	11	100	N/A	14

SOF/VEL, sofosbuvir/velpatasvir; SOF/LED, sofosbuvir/ledipasvir; ELB/GRZ, elbasvir/grazoprevir; GLE/PIB, glecaprevir/pibrentasvir.

**Table 4:** Baseline characteristics of patients treated according to SOC vs shortened treatment.

	Variable	Standard of care N = 18	Shortened treatment N = 11	p value
Demographics	Age (Mean ± SD)	56 ± 16·3	49.4 ± 14.7	0.27
	Female, N (%)	8 (44.4%)	5 (45.5%)	1.00
Fibrosis	METAVIR 3/4, N (%)	5 (27.8%)	2 (18.2%)	0.68
Laboratory at baseline	ALT U/L (Mean ± SD)	65.5 ± 38.7	40.7 ± 12.7	0.05
	Total Bilirubin mg/dL (Mean ± SD)	0.70 ± 0.60	0.56 ± 0.18	0.45
	Albumin g/dL (Mean ± SD)	4.4 ± 0.21	4.6 ± 0.36	0.12
	Creatinine mg/dL (Mean ± SD)	0.83 ± 0.26	0.89 ± 0.38	0.63
	Hb g/dL (Mean ± SD)	13.9 ± 1.2	14.8 ± 1.6	0.11
	PLT 10^6^/mm^6^ (Mean ± SD)	213 ± 69	212 ± 55	0.96
	HCV RNA IU/mL (median and interquartile range)	6.46 (5.92–6.66)	5.88 (5.22–6.35)	0.044

SD, standard deviation; SOC, standard of care; ALT, alanine aminotransferase; Hb, hemoglobin; PLT, platelets; HCV, hepatitis C virus; RNA, ribonucleic acid.

### Treatment outcomes

Twenty eight of the 29 patients (97%) treated with DAA according to the RGT model achieved SVR, one relapsed at week 4 post-treatment and achieved SVR following retreatment with sofosbuvir/velpatasvir/voxilaprevir for 12 weeks. The patient had genotype 3 infection, was non-cirrhotic and treatment naïve, and was treated with SOF/VEL for 6 weeks according to the RGT model (Pt 3, Fig. 2) Sequence analysis at baseline and at week 4 post-treatment did not reveal treatment emergent RAS. During treatment, viral levels < 10 IU/ml were attained in 27/29 patients (93%) at EOT. Two patients showing quantifiable virus levels (21 and 60 IU/mL) at EOT achieved SVR. Eleven patients had viral load below LLoQ at EOT.

### Treatment-time saving

Of the 332 weeks of expected SOC treatment-time for the entire cohort, implementation of the RGT model led to total savings of 46 weeks of DAA treatment. This represents a 14% time saving for the entire cohort. SOF/VEL and ELB/GRZ were associated with the highest percentage of treatment-time saved (17% each) followed by SOF/LED (11%) and GLE/PIB (0%) (Table 3).

## Discussion

In this proof-of-concept pilot study, we show that mathematical modeling of viral kinetics during DAA treatment can be used successfully to individualize treatment duration. Applying an RGT model, based on mathematical modeling, led to a shorter treatment course in 11/29 (38%) patients participating in the study, while maintaining a high proportion of SVR (96.5%) that is consistent with the current performance goal for viral eradication under SOC treatment.

DAA therapy is associated with rapid viral decline over the first days of treatment, with most patients reaching undetectable HCV RNA between week 2–4 of therapy. While SOC is currently based on a fixed duration of treatment in all patients, the high SVR proportions following DAA therapy suggest that cure may be achieved prior to the fixed duration endpoint, at least in a subset of patients. This hypothesis is supported by reports in the literature of patients achieving SVR after stopping treatment prematurely^20–23^ and by multiple clinical trials showing varying degrees of success following ultra-short DAA treatment courses^22,24,25^. In addition, the feasibility of shortening treatment was supported by retrospective studies published by our group over the past several years. By employing mathematical modeling of viral kinetics during early stages of DAA therapy, these studies suggested that the cure boundary can be reached before conventional EOT in the majority of patients treated with SOF-based and other fixed-duration DAA regimens^12,16,17,26^. However, these studies relied on retrospective data to generate population-based rather than individualized models for prediction of TTC, and therefore, the accuracy of such models could not be confirmed. To the best of our knowledge, the current study presents the first attempt to apply a real-time based mathematical model to individualize treatment duration with all-oral HCV DAA therapy.

While elimination of CHC worldwide is theoretically feasible in the DAA era, current drug prices pose a major barrier to achieving this goal in the foreseeable future^8^. Novel strategies aimed at improving the HCV treatment cascade and for optimizing resources allocated for HCV treatment are in urgent need. As demonstrated with the current proof-of-principle study, the use of our RGT modelling approach led to an overall 14%-time savings in treatment duration. This time saving was achieved by using a conservative approach based on three rules; first, the minimal treatment duration allowed was 6 weeks; second, patients whose cure boundaries were predicted to occur between two of our pre-determined cessation timepoints (e.g., 6, 8, 10, or 12 weeks) were assigned to the longer therapy duration; and third, patients having virus levels below LLoQ at day 2 would receive the minimal treatment duration allowed (6 weeks). The latter rule was inspired by results from a previous proof-of-concept study by Lau et al.^27^, showing that ultrashort treatment is possible in patients in whom low viral load is reached at day 2. Modification of the model to allow treatment cessation at the actual timepoint of cure boundary achievement is expected to increase the overall time saving to 23%. Currently, there is a fixed per-treatment price for DAA therapies in developed countries. However, adopting an RGT approach to individualize treatment duration on a larger scale, especially in low-resource settings and in special populations, could expand access to therapy to a greater number of patients under the same budgetary constraints, which could facilitate efforts for global and accessible therapy.

Importantly, all patients (but one) in whom viral load was reduced to < 14 IU/ml at day 14 from initiation of treatment were predicted to reach SVR under shortened duration of DAA therapy. Therefore, we believe that in practice, viral kinetics analysis could be performed in two stages, following collection of blood on day 2 and weeks 1 and 2. The first step would consist of analysis of viral load in all patients at week 2 (day 14). In step 2, HCV RNA measurements at day 2 and week 1 and modeling of all time points only would be performed in patients with viral load < 14 IU/ml at day 14, limiting the cost associated with multiple PCR assays.

The major concern in implementing the RGT model is that it may lead to suboptimal treatment with subsequent failure to achieve SVR. Although the small size of the current study cannot lead to firm conclusions, only one case of viral relapse was recorded in a patient with genotype 3, who was treated with SOF/VEL for 6 weeks. Standard Sanger sequencing did not shown any major, clinically relevant resistant strains. While we could not definitely exclude the existence of minor resistant viral populations due to the lack of formal NGS analysis, we speculate that the likelihood of baseline or treatment emergent RASs is low in this patient population. Considering the fact that in the DAAs era treatment of Gt3 has proven more challenging compared with other HCV genotypes, it is possible that relapse might have occurred even with a full 12-week course of SOF/VEL therapy^28^.

Of the 28 patients achieving SVR, we observed 12 cases in whom this endpoint was accomplished after HCV RNA was either below LLoQ (n = 10) or still quantifiable (n = 2) at EOT. Such events have been previously described in other studies employing highly sensitive platforms for HCV RNA quantification such as the Abbott RealTime HCV assay. In those studies, HCV RNA detectable or quantifiable at EOT, was followed by achievement of SVR in the vast majority of patients^29–31^. Because EOT RNA detection in the interferon era inevitably indicated treatment failure, two explanations have been suggested to account for this DAA-associated phenomenon: 1. The RNA detected represents non-infectious virus particles produced due to DAA-mediated interference of the viral lifecycle^32^. 2. The direct viral inhibition mediated by DAAs better allows for the host immune response to recover its anti-HCV clearance capacity and eliminate any remaining infectious virus^33^. In the current study, HCV quantification was performed by the GeneExpert assay, a real-time PCR platform with sensitivity for HCV detection of LLoQ 10 IU/mL and undetectable limit of 4 IU/mL. As such, it is not surprising we were able to detect the small amounts of HCV RNA at the EOT as others have observed.

This study had several limitations. Due to its small scale, its promising results cannot yet lead to a policy change in HCV management without being validated by a larger scale controlled clinical trial. In addition, due to the small sample size and its exploratory nature, as well as the lack of treatment- experienced patients in this cohort, the study was not powered to detect statistically significant differences in the rates of successful treatment shortening with respect to genotype, fibrosis stage, naïve vs treatment experienced status or different DAA regimens. A multi-center clinical trial designed to address these questions and for validating results of this pilot study, is currently in its initiation stage.

In conclusion, results from this pilot proof-of-principle study show for the first time the utility of RGT for optimizing treatment duration in CHC patients treated with all-oral DAA therapy. Following further validation in a large-scale clinical trial, implementation of an RGT approach for managing CHC at the population level, may lead to significant cost-saving and to improved access to care, especially in resource limited settings.

## Supplementary information

Supplementary information
