# Supplementary material for: Response guided therapy for reducing duration of direct acting antivirals in chronic hepatitis C infected patients: a Pilot study
Source: Sci Rep. 2020 Oct 20;10:17820. doi: 10.1038/s41598-020-74568-x (PMC7575564; doi:10.1038/s41598-020-74568-x)
Supplement: Supplementary file 1 — Supplementary information [file 41598_2020_74568_MOESM1_ESM.docx]

**Supplementary File**

**Response guided therapy for reducing duration of direct acting antivirals in chronic hepatitis C infected patients: A Pilot Study**

Ohad Etzion^1,8*^, Harel Dahari^2*^, David Yardeni^1,8^, Assaf Issachar^3,9^, Anat Nevo-Shor^1,8^, Michal Cohen-Naftaly^3,9^, Yaffa Ashur^4^, Susan L. Uprichard^2^, Orly Sneh Arbib^3,9^, Daniela Munteanu^1,8^, Marius Braun^3,9^, Scott J. Cotler^2^, Naim Abufreha^1,8^, Ayelet Keren-Naus^5^, Yonat Shemer-Avni^5^, Orna Mor^6^, Jayanah Murad^7^, Victor Novack^7^, Amir Shlomai^3,9^

^1^Department of Gastroenterology and Liver Diseases, Soroka University Medical Center, Beer-Sheva, Israel; ^2^The Program for Experimental & Theoretical Modeling, Division of Hepatology, Department of Medicine, Loyola University Chicago, Maywood, IL, USA; ^3^Department of Medicine D and The Liver Institute, Rabin Medical Center, Beilinson Hospital, Petah-Tikva, Israel;^4^ Medical Management Unit, Soroka University Medical Center, Beer-Sheva, Israel;^5^Laboratory of Clinical Virology, Soroka University Medical Center, Beer-Sheva, Israel; ^6^Central Virology Laboratory, Ministry of Health, Sheba Medical Center, Israel;^7^Clinical Research Center, Soroka University Medical Center, Beer-Sheva, Israel; ^8^Faculty of Health Sciences, Ben-Gurion University of the Negev, Beer-Sheva, Israel; ^9^Sackler Faculty of Medicine, Tel-Aviv University, Tel-Aviv, Israel

*These authors share equal contribution

**Full eligibility criteria**

### Inclusion Criteria

1. Signed informed consent
2. Clalit insured patients
3. Female and male over the age of 18
4. Capacity to provide written informed consent
5. HCV RNA Viral Load (VL) larger than 105 IU/mL at screening and on at least one other occasion 6 months or more prior to the most recent HCV RNA test result.
6. HCV genotypes 1a, 1b, 2, 3 or 4
7. Liver fibrosis stage 1-4 as determined by one of the following methods performed within 2 years prior to the screening visit:
8. Fibrotest
9. Transient elastography
10. Liver biopsy using the METAVIR scoring system.
11. Patients must have the following laboratory parameters within 3 months of screening:
12. ALT and AST ≤ x10 the upper limit of normal (ULN)
13. Direct bilirubin ≤ 1.5 the ULN
14. Platelet count ≥70,000
15. Hemoglobin ≥10 mg/dL
16. Albumin ≥3 mg/dL
17. INR ≤ 1.5 x ULN
18. eGFR ≥ 60 mL/min as calculated by the Cockroft-Gault equation.
19. Abdominal ultrasound, C.T or MRI scan showing no evidence of a focal lesion suspicious of hepatocellular carcinoma within 6 months of enrollment.
20. A female patient will be eligible to enter the study if it is confirmed that she is:
21. Not pregnant or nursing
22. Of non-childbearing potential (following hysterectomy, bilateral oophorectomy or post-menopausal)
23. Women of childbearing potential- must have a negative urine pregnancy test at baseline and willing to use an accepted mechanical, medical or surgical birth control method from the day of screening until 90 days from the last dose of study drug.
24. All male participants in the study must agree to consistently and correctly use a condom, while their female partner agrees to use one of the above-mentioned birth control methods from the day of screening until 90 days after the last dose of study drug.
25. Patient must be able to comply with the dosing instructions for the study drug administration and able to complete the study schedule of assessments including all required post-treatment visits.

### Exclusion Criteria

1. Clinical, serologic or histopathological evidence supporting the presence of chronic liver disease other than HCV (Including but not limited to: HBV, HDV or HIV coinfection, non-alcoholic steatohepatitis, alcoholic liver disease, Wilson’s disease, A1AT deficiency and Celiac disease). Workup performed within 6 months of recruitment will be considered sufficient to exclude the above-mentioned conditions (except for A1AT deficiency and Wilson’s disease for which exclusion at any time point qualifies).
2. Current or past history of any of the following:
3. Clinically significant illness (other than HCV) or any other medical disorder that may interfere with patient’s assessment, treatment or compliance with the protocol. Examples include congestive heart disease with moderate to severe left ventricular function and chronic obstructive pulmonary disease requiring chronic corticosteroid therapy.
4. Clinical evidence of decompensated liver disease (e.g. ascites, bleeding esophageal varices, spontaneous bacterial peritonitis, encephalopathy or hepatorenal syndrome.)
5. Child Pugh score higher than 6
6. Gastrointestinal disorder or post-operative condition that may interfere with the absorption of the study drug.
7. Solid organ transplantation
8. Malignancy within 5 years prior to screening with the exception of specific cancers that are entirely cured by surgical resection (basal cell skin cancer etc.) Patients under the evaluation for possible malignancy are not eligible.
9. Any prior treatment with a DAA (protease inhibitors, NS5A inhibitors, NS5B polymerase inhibitors/non-nucleoside polymerase inhibitors)
10. Use of anti-viral medications within 30 days of screening.
11. Chronic use of systemically administered immunosuppressive/immune- modulating medications
12. Clinically relevant substance abuse within 6 months of enrollment. Patient with prior history of drug addiction who are currently maintained on a stable dose of opiate substitutes (naloxone) will be allowed to participate in the study if they can provide documentation of repeated negative toxicology screens from the 6 months prior to screening.
13. Participating in clinical trial 30 days before screening.

**Supplementary Table 1.** Adverse events

| Adverse event | No. of patients | DAA regimen |
| --- | --- | --- |
| Headache | 1 | SOF/LED |
| Nausea | 1 | SOF/LED |
| Weakness | 2 | SOF/VEL; ELB/GRZ |
| Renal impairment | 1 | SOF/VEL |

SOF/VEL, sofosbuvir/velpatasvir; SOF/LED, sofosbuvir/ledipasvir; ELB/GRZ, elbasvir/grazoprevir.
